# Establishing a national high fidelity cadaveric emergency urology simulation course to increase trainee preparedness for independent on-call practice: a prospective observational study

**DOI:** 10.1186/s12909-020-02268-1

**Published:** 2020-10-07

**Authors:** Nicholas Bullock, Thomas Ellul, Suzanne Biers, James Armitage, Sophia Cashman, Krishna Narahari, Oleg Tatarov, Neil Fenn, Pradeep Bose, Jonathan Featherstone, Owen Hughes

**Affiliations:** 1 Division of Cancer and Genetics, Cardiff University School of Medicine, Cardiff, UK; 2 Department of Urology, Cardiff and Vale University Health Board, Cardiff, UK; 3 Department of Urology, Addenbrooke’s Hospital, Cambridge, UK; 4 Department of Urology, Swansea Bay University Health Board, Swansea, UK; 5 Specialist Registrar Education Lead, British Association of Urological Surgeons, London, UK

**Keywords:** Urological emergency, Training, Education, Simulation, Cadaveric

## Abstract

**Background:**

Whilst competence in the management of a wide range of urological emergencies is a requirement for certification in urology, many conditions are uncommon and exposure during training may be limited. This prospective observational study sought to evaluate the feasibility and effectiveness of a standardised cadaveric emergency urology simulation course aimed at improving operative confidence and competence prior to independent on-call practice in the United Kingdom.

**Methods:**

A two-day cadaveric emergency urology simulation course supported by the British Association of Urological Surgeons (BAUS) was implemented at two pilot centres. All delegates that undertook one of the initial series of courses were invited to complete online pre- and post-course questionnaires relating to prior operative experience, documented competence and perceived confidence in being able to perform specific emergency procedures independently. Primary outcome was a self-reported ‘confidence score’ selected from a linear numeric scale ranging from 1 (not at all confident to perform a given procedure independently) to 10 (fully confident). Statistical analysis was undertaken using SPSS Statistics for Mac Version 25 and the paired student’s t-test used to compare mean pre- and post-course scores.

**Results:**

One hundred and four delegates undertook the course during the study period. Of these, 85 (81.7%) completed the pre-course survey and 67 (64.4%) completed the post-course survey, with 61 (58.7%) completing both. The greatest proportion of respondents were Speciality Trainees in Urology of ST5 level or higher (equivalent of Resident/Fellows with 4 or more years of surgical training; *n* = 31, 36.5%). Delegates reported variable pre-course exposure, with most experience reported in loin approach to the kidney (median 10) and least in exploration and packing of a transurethral resection cavity and emergency nephrectomy (median 0). Following course completion, a statistically significant increase in confidence score was observed for each procedure, with the greatest increases seen for shunt for priapism (4.87 to 8.80, *p* < 0.001), ureteric reimplantation (3.52 to 7.33, *p* < 0.001) and primary ureteric anastomosis (3.90 to 7.49, *p* < 0.001).

**Conclusions:**

A standardised high fidelity cadaveric simulation course is feasible and significantly improves the confidence of trainees in performing a wide range of emergency procedures to which exposure is currently limited.

## Background

The assessment and management of emergency cases is an integral component of the workload of Urologists in the United Kingdom (UK) and worldwide. Specialist Trainees (STs; equivalent of Residents and Fellows with more than 2 years of postgraduate surgical training in the US) are therefore required to demonstrate competency to perform a range of emergency procedures independently in order to achieve certification for independent practice from the General Medical Council (GMC; the governing body of medical practitioners in the UK). However, despite this recognition, many of these emergency conditions are uncommon and exposure to both their presentation and surgical treatment during training may be limited. Furthermore, introduction of the European Working Time Directive (EWTD) and changes to the format of surgical training have resulted in significantly fewer working hours between initial medical qualification and completion of specialty training, thereby theoretically further reducing experience [1–3].

In order to evaluate the exposure of UK trainees to uncommon emergency cases, all members of the Specialist Urology Registrars Group (SURG) were sent an email in 2017 inviting them to complete an online survey using the SurveyMonkey platform (SurveyMonkey Inc., San Mateo, USA), with questions relating to number of cases performed and confidence in managing them independently [4]. Ninety four STs with 4 to 7 years of postgraduate surgical training responded to the survey and, despite their relative seniority, reported limited exposure to a number of key emergencies, with 44% having not performed a loin approach to the kidney and 91% having not performed exploration and packing of a transurethral resection (TUR) cavity for bleeding [4]. Collectively this study identified an urgent need to address the current deficit so that trainees are confident and competent in the management of the full spectrum of urological emergencies prior to certification and independent practice.

High fidelity simulation is an established means of acquiring and improving both technical and non-technical skills in all surgical disciplines, including urology [5, 6]. As such, the British Association of Urological Surgeons (BAUS; the professional association of UK urologists) Education Committee have supported development of a cadaveric simulation course to address the aforementioned training deficits and standardise practice across the UK. The aim of this prospective questionnaire based observational study was firstly to confirm current exposure to uncommon urological emergencies and secondly to evaluate the feasibility, quality and impact of initial cadaveric simulation courses run at the Wales Centre for Anatomical Education, Cardiff, and the Evelyn Surgical Training Centre, Cambridge.

## Methods

### Course design and provision

The cadaveric emergency urology course was designed to run to a standard itinerary and implemented at two pilot centres. Each was 2 days in duration, comprising of small group tutorials, facilitated Case Based Discussions (CBDs) and hands-on operating using fresh frozen cadavers, as demonstrated by the summary of course content shown in Table 1. Emphasis was placed on maximal operating time, with a delegate to faculty ratio of 2:1. All faculty were Consultant Urological Surgeons (equivalent of Attending Urologists in the US) with expertise in the procedures covered. Courses were funded via a combination of delegate registration fees, local supplementation and industry sponsorship, with faculty participation being voluntary and unpaid at each centre.

**Table 1:** Summary of course content

Session^a^	Format
Review of anatomy relevant to urological surgery: abdomen, retroperitoneum, pelvis and perineum	Tutorial
Renal trauma: emergency exploration and nephrectomy	Tutorial
Emergency exploration for bleeding, control of major vessels in retroperitoneum and emergency nephrectomy	Practical
Approach to the ureter and management of ureteric injury	Tutorial
Approach to the ureter, anastomotic ureteric repair (end-to-end) and transureteroureterostomy	Practical
Ureteric reimplantation techniques: psoas hitch and Boari flap	Practical
Exploration of pelvis, approach to the bladder and related procedures	Tutorial
Exploration of pelvis, open cystostomy and insertion of suprapubic catheter, repair of bladder rupture, packing of prostatic cavity for bleeding	Practical
Scrotal exploration: testicular fixation and repair of rupture	Tutorial
Scrotal exploration, testicular fixation and repair of rupture	Practical
Andrological emergencies	Tutorial
Penile block, treatment of priapism (including shunt surgery), repair of penile fracture	Practical
Debridement of Fournier’s gangrene and related perineal procedures	Tutorial
Debridement of Fournier’s gangrene, exposure of bulbar urethra, perineal urethrostomy and partial penectomy (if delegate interest)	Practical
Case Based Discussions, completion of assessments and feedback	Tutorial

^a^ Exact content and duration of each session may have differed between courses run at the Cambridge and Cardiff centres

### Data collection

Prior to undertaking the course, delegates were invited to complete a structured online survey (Jisc Online Surveys, Bristol, UK) aimed at establishing pre-existing experience and confidence in the range of procedures covered in the programme, as outlined in Supplementary Material 1. Questions were designed to obtain both objective data relating to delegate experience, based on logbook operative numbers documented on the Intercollegiate Surgical Curriculum Programme electronic portfolio (ISCP ePortfolio; the completion of which is a mandatory requirement of postgraduate surgical training in the UK), and subjective data relating to perceived confidence in being able to perform each procedure independently. Rigid cystoscopy and ureteric stent insertion was included as a positive control to represent the overall emergency operative experience of the cohort, on account of this being the most commonly performed emergency urological procedure [7]. Following course completion, delegates were invited to undertake a second survey aimed at assessing change in confidence and evaluating perceived effectiveness of the course as a whole, as outlined in Supplementary Material 2.

### Primary outcome and statistical analysis

The primary outcome for evaluating course effectiveness was allocation of a self-reported ‘confidence score’ reflecting delegates confidence in performing each procedure independently, selected from a linear numeric scale ranging from 1 (not at all confident) to 10 (fully confident). Linear numeric scales such as this are an established means of evaluating effectiveness in survey based educational research and have previously been utilised to assess a number of metrics, including procedural confidence, following completion of cadaveric simulation training in urology [8]. Statistical analysis was undertaken using SPSS Statistics for Mac Version 25 (IBM Corp, Armonk, USA) and the paired student’s t-test used to compare mean pre- and post-course confidence scores in those delegates that completed both surveys. A significance level of *P* ≤ 0.05 was deemed to denote statistical significance.

## Results

### Delegate demographics

A total of 84 and 20 delegates respectively undertook courses at the Cambridge (5 courses) and Cardiff (2 courses) centres during the study period. Of these, 85 (81.7%) completed the pre-course survey and 67 (64.4%) completed the post-course survey, with 61 (58.7%) completing both. The distribution of grade and training region of those that completed the pre-course survey are given in Table 2. The greatest proportion of delegates were Speciality Trainees in Urology of ST5 level or higher (equivalent of Resident/Fellows with 4 or more years of surgical training, *n* = 31, 36.5%) and the largest number were undertaking training in the East of England (*n* = 20, 23.5%). 8 (9.4%) delegates were of Consultant grade, the majority of whom were employed in Locum Consultant posts.

**Table 2:** Delegate demographics

	Number	%
Training grade		
Foundation Year or Core Surgical Trainee	13	15.3
Specialty Trainee Year 3/4	12	14.1
Specialty Trainee Year 5 or higher	31	36.5
Clinical Fellow	21	24.7
Consultant	8	9.4
UK Training region		
East Midlands	2	2.4
East of England	20	23.5
Kent, Surry & Sussex	10	11.8
London	5	5.9
North East	0	0
North West (North West)	5	5.9
North West (Mersey)	5	5.9
Northern Ireland	1	1.2
Scotland	0	0
Southwest	5	5.9
Thames Valley	0	0
Wessex	3	3.5
West Midlands	4	4.7
Wales	12	14.1
Yorkshire and the Humber	5	5.9
Other	8	9.5

### Pre-course experience in performing specific emergency cases

**Fig. 1:**
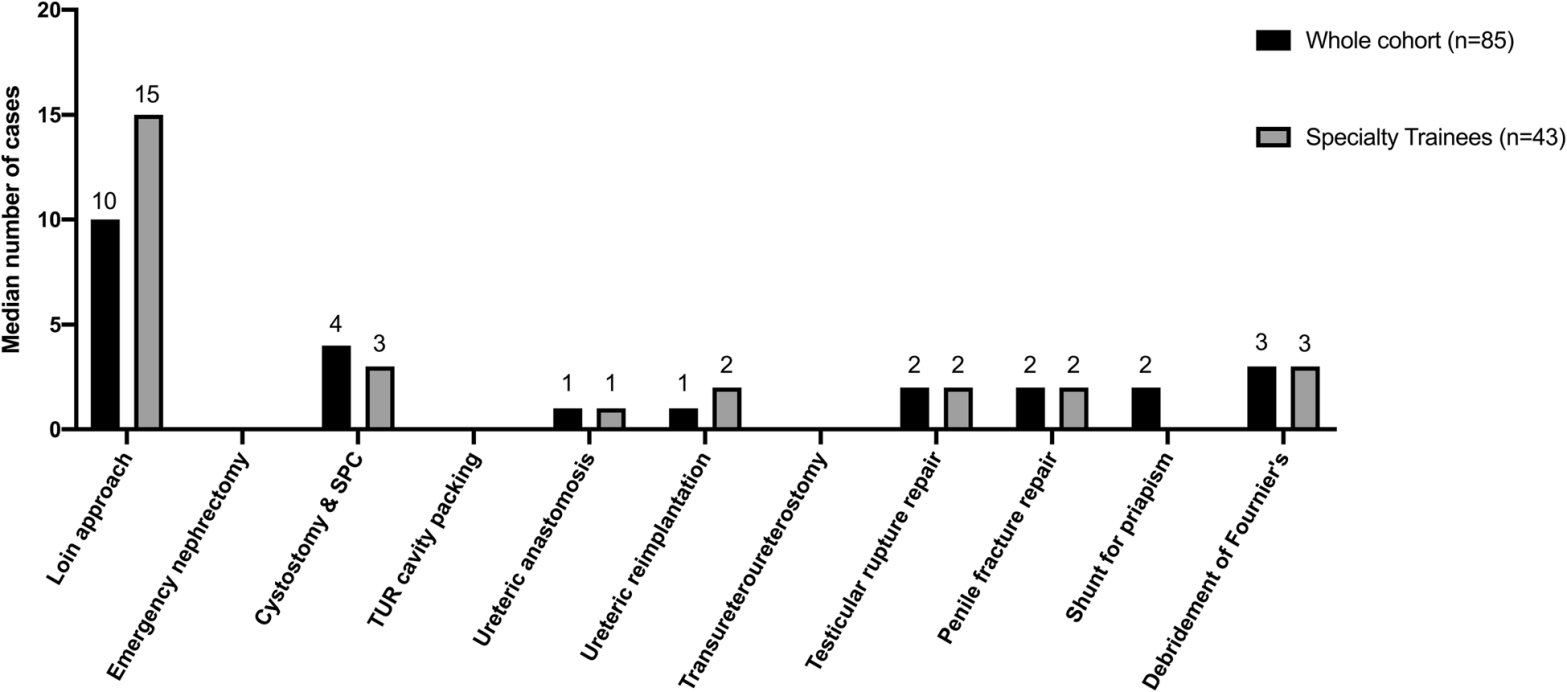
Median total number of each procedure performed by delegates prior to undertaking the course, including all levels of supervision ranging from ‘assisting’ to ‘performed independently’. TUR; transurethral resection, SPC; suprapubic catheter

Figure 1 demonstrates the median number of each emergency procedure performed by delegates prior to undertaking the course, with results displayed for the whole cohort (*n* = 85) as well as the subset of Specialty Trainees (*n* = 43). Delegates reported having performed a median total of 95 rigid cystoscopy and insertion of ureteric stent cases (range 0–1000), collectively indicating sufficient pre-course emergency urology exposure/experience to confirm validity to the results.

Of all the procedures evaluated, delegates reported most experience in loin approach to the kidney (median total 10, range 0–290), open cystostomy and insertion of a suprapubic catheter (median total 4, range 0–110), and debridement of peno-scrotal tissues for Fournier’s (median total 3, range 0–32). However, for all three procedures the majority of cases were reported to be either assisting or performed under supervision. On the contrary, delegates reported very limited pre-course experience in all of the other evaluated procedures. Figures were particularly striking for exploration and packing of a TUR cavity, which is a certification requirement for all surgeons performing endoscopic resections of the prostate. The median number of cases performed by each delegate was 0 for both the whole cohort and Speciality Trainee subset. A similar trend was also seen for primary end-to-end anastomotic repair and ureteric reimplantation, both of which are required for the emergency management of traumatic/iatrogenic ureteric injury, with a median of 1 case each.

### Increase in procedural confidence following course completion

**Fig. 2:**
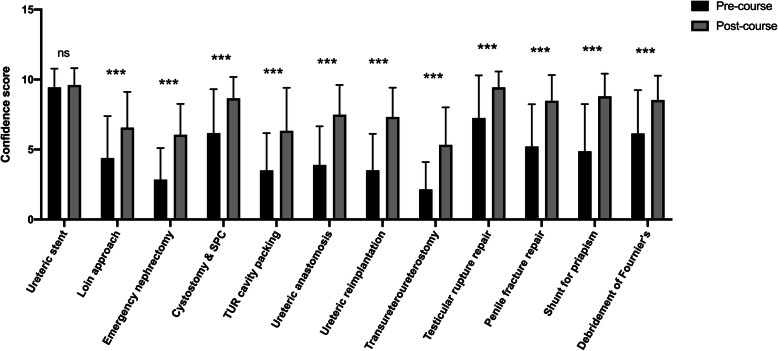
Change in self-reported pre and post course confidence score for each of the procedures covered in the course (*n* = 61). Statistical significance assessed using the paired student’s t-test, ns; not significant, ***; *p* < 0.001

The mean pre- and post-course confidence scores for those delegates that completed both surveys are demonstrated in Fig. 2. As expected, there was no change in confidence in being able to perform rigid cystoscopy and insertion of ureteric stent after course completion (confidence score 9.44 versus 9.61, *p* = 0.077). However, a statistically significant increase in confidence was seen for each procedure covered in the course, with the greatest increase seen for shunt for priapism (confidence score increase from 4.87 to 8.80, *p* < 0.001), ureteric reimplantation (confidence score increase from 3.52 to 7.33, *p* < 0.001) and primary ureteric anastomosis (confidence score increase from 3.90 to 7.49, *p* < 0.001).

### General post-course feedback

**Fig. 3:**
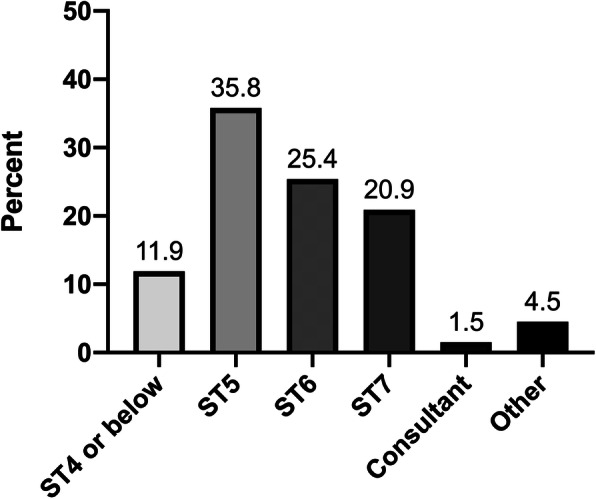
Level of training at which delegates felt it would be appropriate to undertake the course. ST; Specialty Trainee

In the post-course survey delegates were asked a number of general questions relating to the course and their experience, starting with the level of training at which they felt it would be most appropriate. Whilst 8 (11.9%) felt it was appropriate for those of ST4 level or below, the majority indicated that the course would be more suitable for senior trainees, with ST5 being the most commonly indicated level (*n* = 24, 35.8%), as shown in Fig. 3. Overall, all but one delegate (*n* = 66; 98.5%) felt that the course had improved their confidence in approaching uncommon urological emergencies, with all 67 (100%) stating they would recommend attendance to other senior trainees in their region. In addition, 55 (82.1%) felt the course should be mandatory prior to certification.

## Discussion

This study demonstrates that UK trainees currently have limited exposure to a number of uncommon but important urological emergencies, thereby corroborating the findings of the initial survey undertaken by SURG in 2017 [4]. In particular, almost no respondents reported experience in exploration and packing of a TUR cavity for bleeding. This is concerning given that TUR of the prostate, which carries a risk of bleeding requiring transfusion in the region of 2.6–7.1%, remains the standard operative intervention for benign prostatic enlargement (BPE) in the UK, with over 15,000 procedures performed each year [9, 10]. Whilst the majority of bleeding can be managed with conservative, endoscopic or endovascular approaches, open exploration and packing remains an essential last resort and may be life-saving in cases of refractory haemorrhage [11]. Furthermore, although not specifically assessed in this study, trainees are also likely to report limited exposure to open prostate surgery in general given that open operations for both BPE and prostate carcinoma have largely been replaced by endoscopic and robotic assisted laparoscopic techniques.

Similarly, respondents reported very limited pre-existing exposure to the operative management of ureteric injuries using either primary end-to-end anastomotic repair or reimplantation. Whilst ureteric injury is uncommon, accounting for 1–2.5% of all urinary tract trauma, 25% of these are caused by urological surgery and Consultant Urologists are required to manage the full spectrum of cases [12]. Furthermore, recognised intra-operative injury, for example during gynaecological or colorectal surgery, requires the Urologist on-call to be able to perform the necessary surgery immediately and competently under the same anaesthetic.

In General Surgery, emergency patients constitute around 50% of all National Health Service surgical workload, as well as 80% of surgical deaths [13]. This has led to the development of standards by the Royal College of Surgeons of England and a substantial increase in resources, including changes in training and the evolution of ‘Consultant Emergency General Surgeon’ posts in which the practitioner is solely responsible for the management of surgical emergencies [13, 14]. Elsewhere, Emergency General Surgery is recognised as a subspecialty in its own right. For example, in the US doctors are able to train specifically in ‘Acute Care Surgery’ which encompasses trauma, surgical critical care and emergency surgery [15]. In contrast, no such subspecialty of Emergency Urological Surgery is in existence and therefore the majority of Urologists in the UK and worldwide are required to provide an on-call service to manage the full spectrum of emergency conditions. This, taken together with the limited exposure to key emergencies identified in this study, affirms the need to provide high quality and standardised training in emergency urological procedures prior to certification and independent practice.

Simulation has emerged as a valuable means of increasing exposure to, and operative competence in, less common procedures and has proved a valid and evidence based method of teaching both technical and non-technical skills in surgery, as well as a tool for use in recruitment and assessment. Furthermore, simulation based curricula have been shown to flatten the learning curve of complex tasks and train surgeons in rare but critical emergency situations, thereby demonstrating benefits for trainees, trainers and patients alike [16, 17].

As a specialty, Urology has embraced the introduction of simulation, with a large number of models available for training and competency assessment in a variety of skills and procedures. Specific formats range from virtual reality simulators and bench top synthetic models through to live animal models and human cadavers. Whilst each has its own advantages and disadvantages, the largest number of models are available for skills within the field of endourology, owing to the fact that closed cavity procedures lend themselves particularly well to virtual reality and bench top synthetic modelling [18–20]. On the other hand, complex open operations are more difficult to simulate and require use of expensive, less readily available models such as live animals or human cadavers. Given that live animals are not routinely available for surgical training in the UK, human cadavers provide the highest fidelity model for training and experience in open emergency urological surgery and its relevant anatomy. They also have the added benefit that several different procedures, including those performed by different surgical disciplines, can be performed using the same specimen, thereby maximising operative experience that can be gained. Furthermore, cadaveric models have previously been shown to provide both face and content validity for teaching a range of procedures to trainees of all experience levels as part of the British Association of Urological Surgeons (BAUS) Fresh Cadaveric Urology Training Programme [8].

Based on the aforementioned training deficit and strengths of high fidelity cadaveric simulation training, BAUS have supported the development of a specific 2 day emergency urology course utilising fresh frozen cadavers and small group teaching, as outlined in Materials and methods. The results of this study demonstrate this format to be both feasible and effective in increasing the exposure to and confidence of urology trainees in a range of urological emergencies and their operative management. Moreover, all respondents indicated that they would recommend attendance to other senior trainees in their region. Collectively these findings support the notion that the course should be introduced on a national level for all senior trainees, thereby standardising training. Furthermore, we believe that the course structure serves as a model for other countries and regions in which there are similar training deficits and may also have a role in the continuing professional development and revalidation of urologists that have already achieved certification. This may be of particular importance in the aftermath of the worldwide SARS-CoV-2 (COVID-19) pandemic, in which intense pressures facing all heath systems may mandate service provision taking priority over training and education.

This study does however have limitations. Firstly, as training courses such as this are limited by the cost and availability of fresh frozen cadavers, the number of participants was limited due to the number of delegates per course being capped to maintain a target delegate to faculty ratio of 2:1. Another limitation is the reliance on delegate reported data, which may result in recall bias. To account for this questions relating to pre-course experience utilised objective measures such as logbook operative numbers and documented competence level. This enabled delegates to refer back to recorded numbers rather than relying on memory alone. On the contrary, effectiveness of the course was evaluated using a subjective measure of confidence in the form of a self-allocated score on a linear numeric scale. Whilst this enabled direct comparison of pre- and post-course scores, it related only to perceived confidence in being able to perform each procedure independently, rather than objective operative competence. However, due to the cost and limited availability of cadavers, combined with a paucity of validated assessment tools for open emergency urological procedures, undertaking robust pre- and post-course technical skills assessment was not feasible in this study. Future courses that incorporate the assessment of technical skills using validated assessment tools are therefore required to reliably evaluate its impact on operative competence. Delegates attending the course also ranged in training grade, thereby resulting in a broad range of reported pre-course experience. To account for this, median numbers of cases were used to minimise the impact of outliers and rigid cystoscopy and insertion of ureteric stent was included to ensure the pre-course emergency urology exposure/experience of the cohort was sufficient to render the results valid. Finally, as the majority of course faculty were recruited from institutions within the region of the pilot centres, it is possible that the delegates may have known or previously worked with members of the faculty. Whilst this may have resulted in teaching and or reporting bias, it may have also placed delegates at ease and created a more relaxed learning environment, both of which were frequently described as strengths of the course in written feedback.

## Conclusions

This study highlights that UK Specialist Trainees in Urology currently have limited exposure to a number of uncommon urological emergencies for which operative competence is required for certification. However, a specifically designed two-day cadaveric simulation course is feasible and provides trainees with the opportunity to perform complex emergency procedures in a safe and supervised setting. In doing so it significantly improves trainee confidence in performing a wide range of emergency procedures independently and supports implementation on a national basis to address current deficits and standardise training.

## Supplementary information

**Additional file 1: Supplementary Material 1.** Pre-course questionnaire

**Additional file 2: Supplementary Material 2.** Post-course questionnaire

## Data Availability

All data generated or analysed during this study are included in this published article and its supplementary information files.

## Abbreviations

BAUS: British Association of Urological Surgeons
BPE: Benign prostatic enlargement
CBD: Case Based Discussion
EWTD: European Working Time Directive
GMC: General Medical Council
ISCP: Intercollegiate Surgical Curriculum Programme
NHS: National Health Service
SPC: Suprapubic catheter
ST: Specialist Trainee
SURG: Specialist Urology Registrars Group
TUR: Transurethral resection
TUU: Transureteroureterostomy
UK: United Kingdom
US: United States
