# Supplementary material for: Establishing a national high fidelity cadaveric emergency urology simulation course to increase trainee preparedness for independent on-call practice: a prospective observational study
Source: BMC Med Educ. 2020 Oct 7;20:349. doi: 10.1186/s12909-020-02268-1 (PMC7540436; doi:10.1186/s12909-020-02268-1)
Supplement: Supplementary file 1 — Additional file 1: Supplementary Material 1. Pre-course questionnaire [file 12909_2020_2268_MOESM1_ESM.docx]

Establishing a national high fidelity cadaveric emergency urology course to increase trainee preparedness for independent on-call practice in the United Kingdom

Supplementary Material 1: Pre-course questionnaire

*Question 1*

What is your current level of training?

1. ST5
2. ST6
3. ST7
4. Other – please specify

*Question 2*

In what Deanery are you undertaking your training?

1. East Midlands
2. East of England
3. North East
4. North West (North West)
5. North West (Mersey)
6. Scotland
7. London
8. Kent, Surrey & Sussex
9. Southwest
10. Thames Valley
11. Wessex
12. West Midlands
13. Yorkshire and the Humber
14. Northern Ireland
15. Wales
16. Other – please specify

*Question 3*

At which centre are you undertaking the course?

1. Cardiff
2. Cambridge

*Question 4*

Without yet attending the course, how much do you think is a reasonable price for a cadaveric course such as this?

1. Less than £100
2. £100-200
3. £200-300
4. £300-400
5. More than £400

*Question 5*

*5.1 Cystoscopy and ureteric stent insertion*

Please indicate the number of the above procedure that you have performed to date (as stratified according to Intercollegiate Surgical Curriculum Programme (ISCP) eLogbook supervision category):

1. Assisted
2. Supervised - trainer scrubbed
3. Supervised – trainer unscrubbed but in theatre
4. Performed

Please indicate the maximum competence level documented as a Procedure Based Assessment (PBA) or Direct Observation of Procedural Skills (DOPS) on ISCP:

1. Level 1
2. Level 2
3. Level 3
4. Level 4
5. I have not undertaken a PBA or DOPS undertaken for this procedure

On a scale of 1-10, please indicate your confidence in being able to perform this procedure independently at your current level of training (1 = not confident, 10 = fully confident):

1. Scale from 1 - 10

*5.2 Loin approach to the kidney and retroperitoneum*

Please indicate the number of the above procedure that you have performed to date (as stratified according to Intercollegiate Surgical Curriculum Programme (ISCP) eLogbook supervision category):

1. Assisted
2. Supervised - trainer scrubbed
3. Supervised – trainer unscrubbed but in theatre
4. Performed

Please indicate the maximum competence level documented as a Procedure Based Assessment (PBA) or Direct Observation of Procedural Skills (DOPS) on ISCP:

1. Level 1
2. Level 2
3. Level 3
4. Level 4
5. I have not undertaken a PBA or DOPS undertaken for this procedure

On a scale of 1-10, please indicate your confidence in being able to perform this procedure independently at your current level of training (1 = not confident, 10 = fully confident):

1. Scale from 1 - 10

*5.3 Emergency nephrectomy*

Please indicate the number of the above procedure that you have performed to date (as stratified according to Intercollegiate Surgical Curriculum Programme (ISCP) eLogbook supervision category):

1. Assisted
2. Supervised - trainer scrubbed
3. Supervised – trainer unscrubbed but in theatre
4. Performed

Please indicate the maximum competence level documented as a Procedure Based Assessment (PBA) or Direct Observation of Procedural Skills (DOPS) on ISCP:

1. Level 1
2. Level 2
3. Level 3
4. Level 4
5. I have not undertaken a PBA or DOPS undertaken for this procedure

On a scale of 1-10, please indicate your confidence in being able to perform this procedure independently at your current level of training (1 = not confident, 10 = fully confident):

1. Scale from 1 - 10

*5.4 Open cystotomy and suprapubic catheter insertion*

Please indicate the number of the above procedure that you have performed to date (as stratified according to Intercollegiate Surgical Curriculum Programme (ISCP) eLogbook supervision category):

1. Assisted
2. Supervised - trainer scrubbed
3. Supervised – trainer unscrubbed but in theatre
4. Performed

Please indicate the maximum competence level documented as a Procedure Based Assessment (PBA) or Direct Observation of Procedural Skills (DOPS) on ISCP:

1. Level 1
2. Level 2
3. Level 3
4. Level 4
5. I have not undertaken a PBA or DOPS undertaken for this procedure

On a scale of 1-10, please indicate your confidence in being able to perform this procedure independently at your current level of training (1 = not confident, 10 = fully confident):

1. Scale from 1 - 10

*5.5 Exploration and packing of a TUR cavity for bleeding*

Please indicate the number of the above procedure that you have performed to date (as stratified according to Intercollegiate Surgical Curriculum Programme (ISCP) eLogbook supervision category):

1. Assisted
2. Supervised - trainer scrubbed
3. Supervised – trainer unscrubbed but in theatre
4. Performed

Please indicate the maximum competence level documented as a Procedure Based Assessment (PBA) or Direct Observation of Procedural Skills (DOPS) on ISCP:

1. Level 1
2. Level 2
3. Level 3
4. Level 4
5. I have not undertaken a PBA or DOPS undertaken for this procedure

On a scale of 1-10, please indicate your confidence in being able to perform this procedure independently at your current level of training (1 = not confident, 10 = fully confident):

1. Scale from 1 - 10

*5.6 Primary / end-to-end anastomotic repair of ureteric injury*

Please indicate the number of the above procedure that you have performed to date (as stratified according to Intercollegiate Surgical Curriculum Programme (ISCP) eLogbook supervision category):

1. Assisted
2. Supervised - trainer scrubbed
3. Supervised – trainer unscrubbed but in theatre
4. Performed

Please indicate the maximum competence level documented as a Procedure Based Assessment (PBA) or Direct Observation of Procedural Skills (DOPS) on ISCP:

1. Level 1
2. Level 2
3. Level 3
4. Level 4
5. I have not undertaken a PBA or DOPS undertaken for this procedure

On a scale of 1-10, please indicate your confidence in being able to perform this procedure independently at your current level of training (1 = not confident, 10 = fully confident):

1. Scale from 1 - 10

*5.7 Ureteric reimplantation with psoas hitch or Boari flap*

Please indicate the number of the above procedure that you have performed to date (as stratified according to Intercollegiate Surgical Curriculum Programme (ISCP) eLogbook supervision category):

1. Assisted
2. Supervised - trainer scrubbed
3. Supervised – trainer unscrubbed but in theatre
4. Performed

Please indicate the maximum competence level documented as a Procedure Based Assessment (PBA) or Direct Observation of Procedural Skills (DOPS) on ISCP:

1. Level 1
2. Level 2
3. Level 3
4. Level 4
5. I have not undertaken a PBA or DOPS undertaken for this procedure

On a scale of 1-10, please indicate your confidence in being able to perform this procedure independently at your current level of training (1 = not confident, 10 = fully confident):

1. Scale from 1 - 10

*5.8 Transureteroureterostomy (TUU)*

Please indicate the number of the above procedure that you have performed to date (as stratified according to Intercollegiate Surgical Curriculum Programme (ISCP) eLogbook supervision category):

1. Assisted
2. Supervised - trainer scrubbed
3. Supervised – trainer unscrubbed but in theatre
4. Performed

Please indicate the maximum competence level documented as a Procedure Based Assessment (PBA) or Direct Observation of Procedural Skills (DOPS) on ISCP:

1. Level 1
2. Level 2
3. Level 3
4. Level 4
5. I have not undertaken a PBA or DOPS undertaken for this procedure

On a scale of 1-10, please indicate your confidence in being able to perform this procedure independently at your current level of training (1 = not confident, 10 = fully confident):

1. Scale from 1 - 10

*5.9 Scrotal exploration and repair of testicular rupture*

Please indicate the number of the above procedure that you have performed to date (as stratified according to Intercollegiate Surgical Curriculum Programme (ISCP) eLogbook supervision category):

1. Assisted
2. Supervised - trainer scrubbed
3. Supervised – trainer unscrubbed but in theatre
4. Performed

Please indicate the maximum competence level documented as a Procedure Based Assessment (PBA) or Direct Observation of Procedural Skills (DOPS) on ISCP:

1. Level 1
2. Level 2
3. Level 3
4. Level 4
5. I have not undertaken a PBA or DOPS undertaken for this procedure

On a scale of 1-10, please indicate your confidence in being able to perform this procedure independently at your current level of training (1 = not confident, 10 = fully confident):

1. Scale from 1 - 10

*5.10 Penile fracture repair*

Please indicate the number of the above procedure that you have performed to date (as stratified according to Intercollegiate Surgical Curriculum Programme (ISCP) eLogbook supervision category):

1. Assisted
2. Supervised - trainer scrubbed
3. Supervised – trainer unscrubbed but in theatre
4. Performed

Please indicate the maximum competence level documented as a Procedure Based Assessment (PBA) or Direct Observation of Procedural Skills (DOPS) on ISCP:

1. Level 1
2. Level 2
3. Level 3
4. Level 4
5. I have not undertaken a PBA or DOPS undertaken for this procedure

On a scale of 1-10, please indicate your confidence in being able to perform this procedure independently at your current level of training (1 = not confident, 10 = fully confident):

1. Scale from 1 - 10

*5.11 Shunt for priapism*

Please indicate the number of the above procedure that you have performed to date (as stratified according to Intercollegiate Surgical Curriculum Programme (ISCP) eLogbook supervision category):

1. Assisted
2. Supervised - trainer scrubbed
3. Supervised – trainer unscrubbed but in theatre
4. Performed

Please indicate the maximum competence level documented as a Procedure Based Assessment (PBA) or Direct Observation of Procedural Skills (DOPS) on ISCP:

1. Level 1
2. Level 2
3. Level 3
4. Level 4
5. I have not undertaken a PBA or DOPS undertaken for this procedure

On a scale of 1-10, please indicate your confidence in being able to perform this procedure independently at your current level of training (1 = not confident, 10 = fully confident):

1. Scale from 1 - 10

*5.12 Debridement of peno-scrotal tissues for Fournier’s*

Please indicate the number of the above procedure that you have performed to date (as stratified according to Intercollegiate Surgical Curriculum Programme (ISCP) eLogbook supervision category):

1. Assisted
2. Supervised - trainer scrubbed
3. Supervised – trainer unscrubbed but in theatre
4. Performed

Please indicate the maximum competence level documented as a Procedure Based Assessment (PBA) or Direct Observation of Procedural Skills (DOPS) on ISCP:

1. Level 1
2. Level 2
3. Level 3
4. Level 4
5. I have not undertaken a PBA or DOPS undertaken for this procedure

On a scale of 1-10, please indicate your confidence in being able to perform this procedure independently at your current level of training (1 = not confident, 10 = fully confident):

1. Scale from 1 - 10
