# Supplementary material for: Establishing a national high fidelity cadaveric emergency urology simulation course to increase trainee preparedness for independent on-call practice: a prospective observational study
Source: BMC Med Educ. 2020 Oct 7;20:349. doi: 10.1186/s12909-020-02268-1 (PMC7540436; doi:10.1186/s12909-020-02268-1)
Supplement: Supplementary file 2 — Additional file 2: Supplementary Material 2. Post-course questionnaire [file 12909_2020_2268_MOESM2_ESM.docx]

Establishing a national high fidelity cadaveric emergency urology course to increase trainee preparedness for independent on-call practice in the United Kingdom

Supplementary Material 2: Post-course questionnaire

*Question 1:*

Having now attended the course, on a scale of 1-10, please indicate your confidence in being able to perform the following procedures independently

(1 = not confident, 10 = fully confident):

*1.1 Cystoscopy and ureteric stent insertion*

1. Scale from 1 - 10

*1.2 Loin approach to the kidney and retroperitoneum*

1. Scale from 1 - 10

*1.3 Emergency nephrectomy*

1. Scale from 1 - 10

*1.4 Open cystotomy and suprapubic catheter insertion*

1. Scale from 1 - 10

*1.5 Exploration and packing of a TUR cavity for bleeding*

1. Scale from 1 - 10

*1.6 Primary / end-to-end anastomotic repair of ureteric injury*

1. Scale from 1 - 10

*1.7 Ureteric reimplantation with psoas hitch or Boari flap*

1. Scale from 1 - 10

*1.8 Transureteroureterostomy*

1. Scale from 1 - 10

*1.9 Scrotal exploration and repair of testicular rupture*

1. Scale from 1 - 10

*1.10 Penile fracture repair*

1. Scale from 1 - 10

*1.11 Shunt for priapism*

1. Scale from 1 - 10

*1.12 Debridement of peno-scrotal tissues for Fournier’s*

1. Scale from 1 - 10

*Question 2:*

Having now attended the course, how much do you think is a reasonable price for a cadaveric course such as this?

1. Less than £100
2. £100-200
3. £200-300
4. £300-400
5. More than £400

*Question 3:*

Having now attended the course, at what level of training do you think it should be undertaken?

1. ST5
2. ST6
3. ST7
4. Other – please specify

*Question 4:*

Overall, do you feel this course has improved your confidence in approaching complex urological emergencies?

1. Yes
2. No
3. Not sure

*Question 5:*

Would you recommend this course to other senior trainees working in your region?

1. Yes
2. No
3. Not sure

*Question 6:*

Do you think this course should be mandatory for all senior trainees in the United Kingdom prior to obtaining CCT?

1. Yes
2. No
3. Not sure

*Question 7:*

Would you be prepared for a proportion of your annual study leave budget to be used to pay for attendance on this course?

1. Yes
2. No
3. Not sure

*Question 8:*

Do you think this course should be centrally funded?

1. Yes
2. No
3. Not sure

*Question 9:*

Do you think any of the stations are unnecessary and could be dropped from the course? If so, please indicate which ones (select as many as required):

1. Loin approach to the kidney and retroperitoneum
2. Emergency nephrectomy
3. Open cystotomy and suprapubic catheter insertion
4. Exploration and packing of a TUR cavity for bleeding
5. Primary / end-to-end anastomotic repair of ureteric injury
6. Ureteric reimplantation with psoas hitch or Boari flap
7. Transureteroureterostomy
8. Scrotal exploration and repair of testicular rupture
9. Penile fracture repair
10. Shunt for priapism
11. Debridement of peno-scrotal tissues for Fournier’s

*Question 10:*

Are there any other procedures that should be covered in the course?

1. Free text response

*Question 11:*

What aspects of the course worked well?

1. Free text response

*Question 12:*

What aspects of the course could be improved for future years?

1. Free text response
